# The potential crosstalk genes and molecular mechanisms between systemic lupus erythematosus and periodontitis

**DOI:** 10.3389/fgene.2025.1527713

**Published:** 2025-04-16

**Authors:** Kai Zhao, Xiaolong Li, Qingmiao Zhu, Mengyu Zhu, Jinge Huang, Ting Zhao

**Affiliations:** ^1^ College of Basic Medical Science, Zhejiang Chinese Medical University, Hangzhou, China; ^2^ The First Affiliated Hospital of Zhejiang Chinese Medical University, Hangzhou, China; ^3^ Key Laboratory of Chinese Medicine Rheumatology of Zhejiang Province, Research Institute of Chinese Medical Clinical Foundation and Immunology, College of Basic Medical Science, Zhejiang Chinese Medical University, Hangzhou, China

**Keywords:** systemic lupus erythematosus, periodontitis, PI3K-Akt signaling pathway, VCAN, LY96

## Abstract

**Background:**

Several studies have demonstrated an increased risk of periodontitis (PD) among patients diagnosed with systemic lupus erythematosus (SLE). However, the underlying common mechanism between them remains incompletely understood. Accordingly, the aim of this study is to examine diagnostic biomarkers and potential therapeutic targets for SLE and PD by leveraging publicly accessible microarray datasets and transcriptome analysis.

**Method:**

Datasets pertaining to SLE and PD were retrieved from the Gene Expression Omnibus (GEO) database, and subsequently analyzed for differentially expressed genes (DEGs). Key gene modules were identified through weighted gene co-expression network analysis (WGCNA), and shared genes were obtained by overlapping key genes between DEGs and WGCNA. These shared genes were subsequently subjected to Gene Ontology (GO) and Kyoto Encyclopedia of Genes and Genomes (KEGG) pathway enrichment analyses, leading to the establishment of a Protein-Protein Interaction (PPI) network. Random forest (RF) and Least Absolute Shrinkage and Selection Operator (Lasso) regression were employed to identify key hub genes. Receiver operating characteristic (ROC) curves were generated using a new validation dataset to evaluate the performance of candidate genes. Finally, levels of immune cell infiltration in SLE and PD were assessed using CIBERSORTx.

**Results:**

A total of 50 core genes were identified between the genes screened by WGCNA and DEGs. Functional enrichment analysis revealed that these genes are primarily associated with the PI3K-Akt and B-cell receptor signaling pathways. Additionally, using machine learning algorithms and ROC curve analysis, a total of 8 key genes (PLEKHA1, CEACAM1, TNFAIP6, TCN2, GLDC, GNG7, LY96, VCAN) were identified Finally, immune infiltration analysis highlighted the significant roles of neutrophils, monocytes, plasma cells, and gammadelta T cells (γδ T cells) in the pathogenesis of both SLE and PD.

**Conclusion:**

This study identifies 8 hub genes that could potentially serve as diagnostic markers for both SLE and PD, highlighting the importance of VCAN and LY96 in diagnosis. Moreover, the involvement of the PI3K-Akt signaling pathway in both diseases suggests its significant role. These identified key genes and signaling pathways lay the groundwork for deeper comprehension of the interplay between SLE and PD.

## 1 Introduction

Systemic lupus erythematosus (SLE) is a complex inflammatory autoimmune disease characterized by the interplay of genetic, immunological, and environmental factors (Akhil et al., 2023). The multi-system involvement of SLE results in a broad spectrum of clinical manifestations, often affecting the skin, joints, kidneys, cardiovascular, and respiratory systems (Fava and Petri, 2019; Ameer et al., 2022). In recent years, there has been growing attention to oral health complications in SLE patients, including temporomandibular joint dysfunction, tooth loss, and oral mucosal lesions (Aceves-Avila et al., 2013; Benli et al., 2021). Notably, the incidence of dental caries is significantly higher in SLE patients than in the general population, closely linked to reduced salivary secretion and alterations in the oral microbiome (Loyola Rodriguez et al., 2016). These oral health issues not only diminish the quality of life but also may exacerbate the systemic burden of the disease (Corrêa et al., 2018).

Periodontitis (PD) is a widespread chronic inflammatory disease, with a global prevalence estimated between 20% and 50% (Nazir, 2017). The pathophysiology of PD is primarily driven by bacterial infection, leading to persistent inflammation and the destruction of periodontal tissues (Sedghi et al., 2021). The progression of PD includes gingival inflammation, formation of periodontal pockets, bone resorption, and eventual tooth loss (Papapanou et al., 2018; Tonetti et al., 2018). PD is closely associated with several systemic diseases, including cardiovascular diseases, diabetes, rheumatoid arthritis, and SLE (Rutter-Locher et al., 2017; Baima et al., 2022; Kobayashi and Bartold, 2023). Among these, the relationship between SLE and PD has received particular attention. Epidemiological studies indicate that SLE patients are at a significantly increased risk of developing PD (Hussain et al., 2022). Clinical studies further reveal a bidirectional influence between SLE and PD. One study demonstrated that periodontal treatment in SLE patients significantly reduced disease activity, suggesting that effective management of periodontitis may positively impact the systemic condition of SLE (Fabbri et al., 2014).

Genetic studies reveal that SLE and PD share specific pathogenic genes and signaling pathways. For instance, polymorphisms in the Fcγ receptor gene play a crucial role in both diseases (Kobayashi et al., 2003; Kobayashi et al., 2007). Certain Fcγ receptor alleles in SLE patients are associated with more severe periodontal destruction, highlighting the importance of genetic susceptibility in the shared pathogenesis of these two diseases (Kobayashi et al., 2003). Additionally, Toll-like receptor (TLR) signaling pathways are abnormally activated in both SLE and PD, further suggesting an immunopathological link between them (Marques et al., 2016; Wallet et al., 2018). From a microbiological standpoint, immune dysfunction in SLE patients leads to an imbalance in the oral microbiome, facilitating the overgrowth of periodontal pathogens (Bagavant et al., 2018; Pessoa et al., 2019). Specifically, Porphyromonas gingivalis is believed to play a central role in the pathogenesis of both PD and SLE. Studies have shown that the abundance of this pathogen is significantly higher in the oral cavities of SLE patients and is correlated with the severity of periodontal tissue destruction (Graves et al., 2019). Furthermore, elevated levels of pro-inflammatory cytokines (such as TNF-α and IL-6) in SLE patients exacerbate periodontal inflammation, resulting in more severe damage to periodontal tissues (Martínez-García and Hernández-Lemus, 2021). The existing evidence strongly supports a close biological connection between SLE and PD. Further exploration of the shared mechanisms underlying these two diseases could not only enhance the understanding of their pathogenesis but also provide novel therapeutic approaches for their combined management.

In this study, we comprehensively analyzed gene expression datasets for both SLE and PD obtained from the GEO database. The identification of shared genes between SLE and PD was achieved through the detection of DEGs and WGCNA of the dataset. Subsequently, we conducted GO and KEGG analyses on the identified genes, followed by the construction of PPI networks. To substantiate our findings, we employed machine learning algorithms, specifically RF and LASSO, to ascertain common core genes possessing the utmost diagnostic significance in both afflictions. Finally, we conducted immune cell infiltration analysis to examine the differences in immune cell composition between SLE and PD patients, and assessed whether these differences correlate with the expression levels of shared core genes. Through this comprehensive bioinformatics analysis, we aim to offer new insights and perspectives for the clinical management of SLE concurrent with PD by enhancing our understanding of the biological connections between SLE and PD.

## 2 Materials and methods

### 2.1 Data download

**FIGURE 1:**
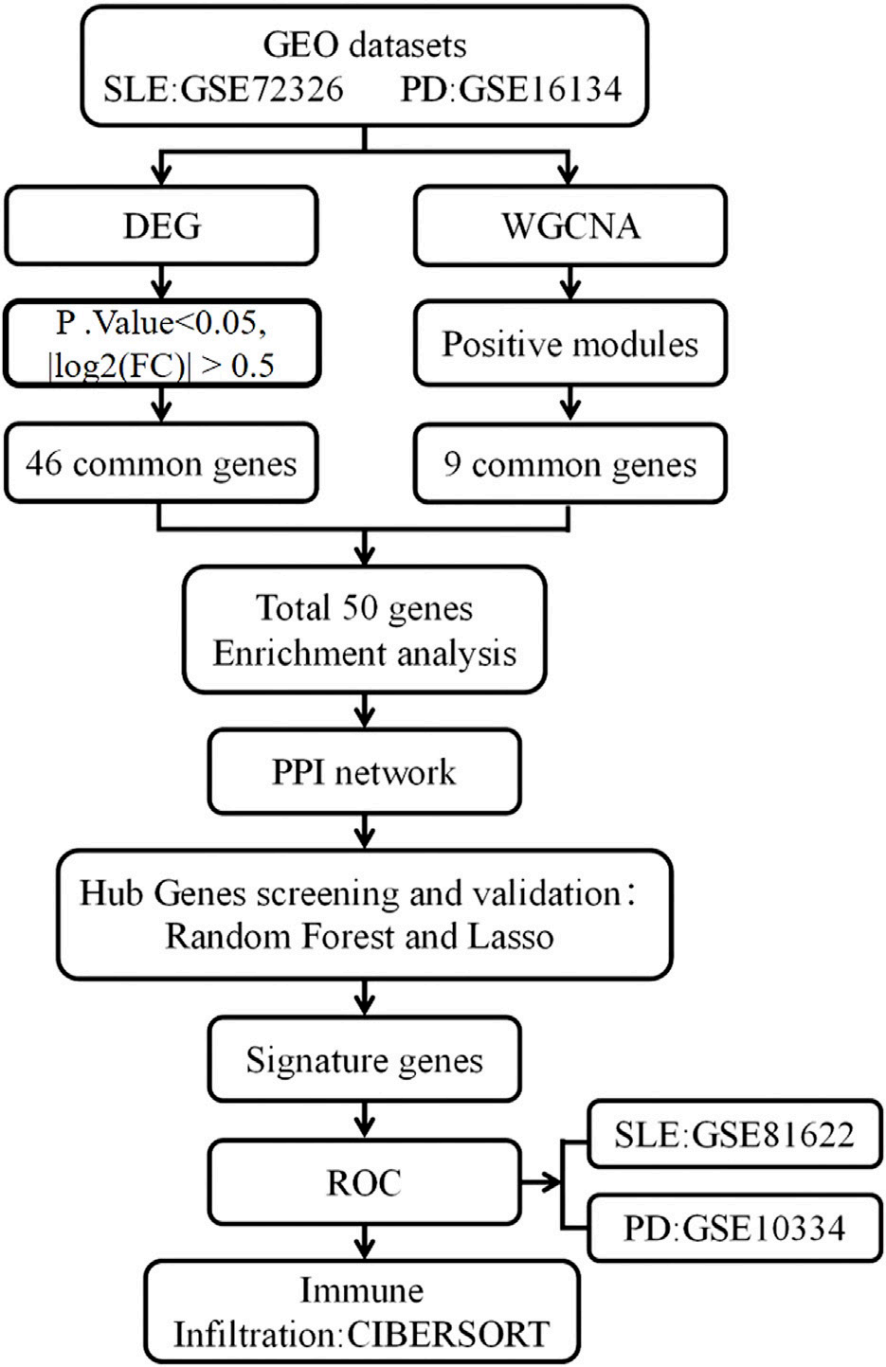
Research study flowchart.

The research process is illustrated in Figure 1. Data sets GSE72326, GSE16134, GSE81622, and GSE10334, relevant to SLE and PD, were obtained from the GEO database (https://www.ncbi.nlm.nih.gov/geo/) (Barrett et al., 2013). The GSE72326 dataset comprised 157 samples from individuals with SLE and 20 healthy controls. GSE16134 encompassed 241 samples from individuals with PD alongside 69 healthy controls. Additionally, we designated GSE81622 and GSE10334 as validation datasets, with GSE81622 comprising 30 SLE samples and 25 healthy controls, while GSE10334 included 183 samples from individuals with PD and 64 healthy controls.

To ensure consistency across the different platforms of these datasets, we performed several preprocessing steps. Initially, all gene identifiers were converted to gene symbols. Subsequently, we assessed the normality of the data distribution. For datasets exhibiting skewed distributions, a log2 transformation was applied to normalize the data. These steps were critical in minimizing platform-specific biases and ensuring the comparability of the datasets.

### 2.2 Differential gene expression analysis

We employed the R software package “Limma” to conduct an analysis of distinctions between the datasets GSE72326 and GSE16134. Differential gene screening was performed using thresholds of P. Value < 0.05 and |log2FC| > 0.5 (Lv et al., 2022). Volcano plots were generated using R software to visualize these DEGs. Additionally, the shared DEGs between SLE and PD were visualized using a Venn diagram.

### 2.3 WGCNA analysis

We conducted WGCNA on the datasets GSE72326 and GSE16134. The function “pickSoftThreshold” was utilized to screen gene modules (Xia et al., 2023). The top 25% of genes ranked by absolute median deviation were chosen for WGCNA. After eliminating missing values and outliers, we constructed the adjacency matrix using the soft thresholding method with a scale-free R^2 > 0.8 criterion. Subsequently, the adjacency matrix was transformed into a topological overlap matrix. Genes were clustered based on their topological overlap matrix using the average linkage hierarchical clustering method. We defined key modules requiring a minimum of 30 genes and a cut height of 0.25. Finally, Pearson correlation analysis was conducted to assess the relationship between these modules and disease. Core modules with the highest Pearson coefficients were then selected for further analysis.

### 2.4 Enrichment analysis and PPI network construction

After integrating the WGCNA results with DEGs, we conducted GO term annotation and KEGG pathway analysis using Metascape (http://metascape.org/gp/index.html#/main/step1). The GO enrichment analysis was categorized into three main domains: BP, CC, and MF. Additionally, KEGG pathway analysis was performed to identify significant biological pathways associated with DEGs. The visualization of GO and KEGG enrichment results was generated using the Bioinformatics Online Platform (http://www.bioinformatics.com.cn/).

We integrated WGCNA results with DEGs and uploaded them to the STRING database for PPI network construction. The required interaction score was set to >0.4. Subsequently, we visualized the results with Cytoscape software and identified the central gene using the CytoHubba plugin within Cytoscape (http://www.cytoscape.org).

### 2.5 Machine learning-based candidate gene selection

We applied the LASSO algorithm, a regression method for variable selection and predictive performance enhancement, along with the RF algorithm to identify hub genes. The analyses were conducted using the “glmnet” and “randomForest” packages in R (Díaz-Uriarte and Alvarez de Andrés, 2006; Garcia-Magariños et al., 2010). In the LASSO model, the regularization parameter (lambda) was optimized by selecting the value that minimized the mean cross-validation error, while the RF model was constructed with 1,000 decision trees. Subsequently, we performed ROC curve analysis using the “rms” package to evaluate the overlapping genes identified by both LASSO and RF. Genes with an AUC exceeding 0.6 were considered potential candidate biomarkers.

### 2.6 Construction and validation of line plots for candidate biomarker

In order to further validate the potential of the selected genes for diagnosis, we assessed the ROC curves of these overlapping genes using the GSE81622 and GSE10334 datasets. Genes with an Area Under the Curve (AUC) value exceeding 0.6 were considered to have significant diagnostic utility.

### 2.7 Immune cell infiltration analysis

We employed the CIBERSORT algorithm to evaluate the distribution of immune cells between disease and normal samples (Newman et al., 2015). Specifically, this study investigated the distribution of 22 immune cell types in samples from GSE72326 and GSE16134, with significance determined by p-values < 0.05. Additionally, Pearson correlation analysis was conducted between differentially coexpressed genes and immunoinfiltrating cells. Data visualization utilized the vioplot and pheatmap R packages.

## 3 Results

### 3.1 Analysis of differential gene expression

**FIGURE 2:**
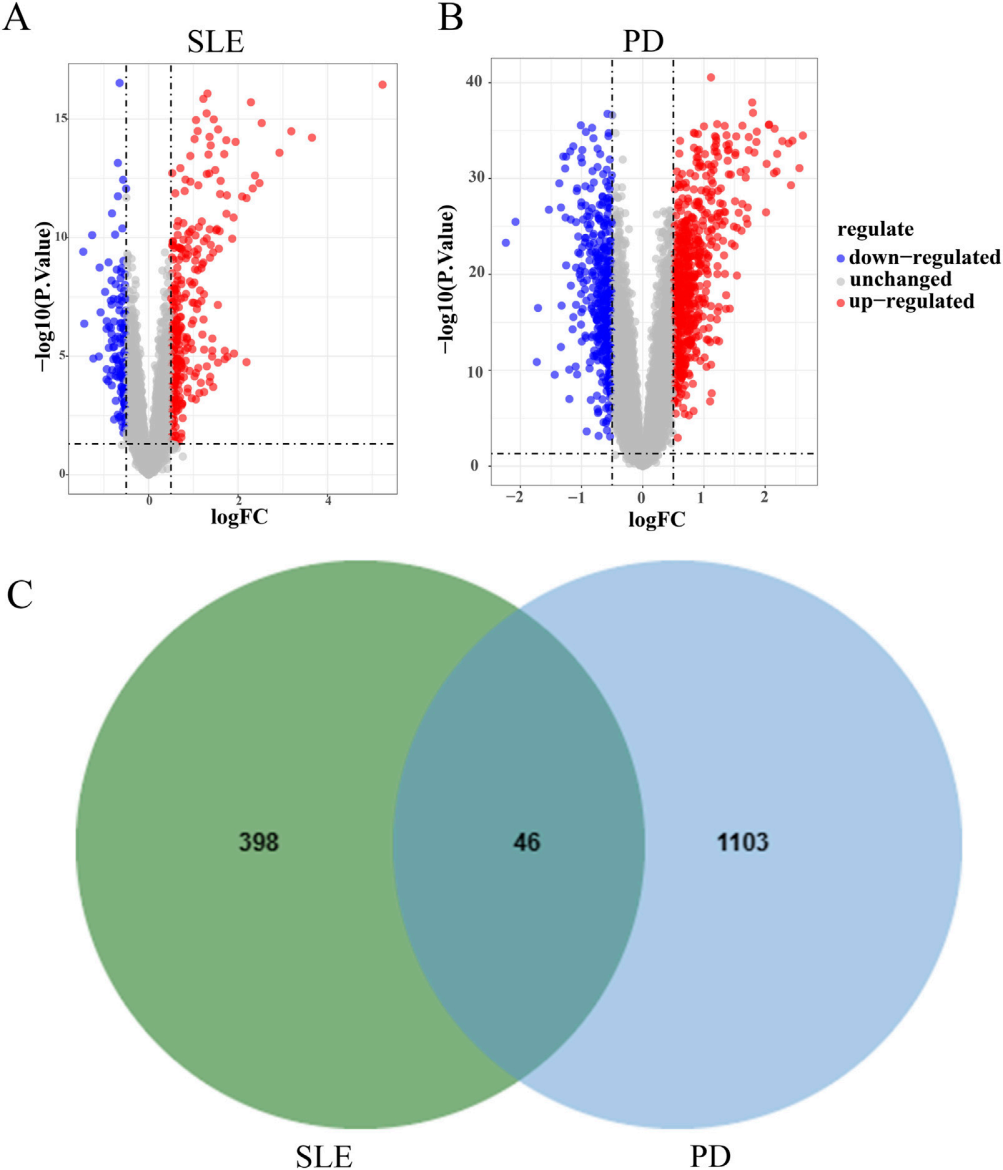
Analysis of differential gene expression. **(A, B)** Volcano plots illustrated the presence of DEGs in datasets GSE72326 and GSE16134. **(C)** Overlapping DEGs of GSE72326 and GSE16134.

DEGs in datasets SLE GSE72326 and PD GSE16134 underwent analysis via the limma R package. Visualization via volcano plots depicted the DEGs. In the SLE dataset GSE72326, 444 DEGs were identified, comprising 302 genes showing upregulation and 142 genes exhibiting downregulation (Figure 2A). Dataset PD GSE16134 yielded 1,149 DEGs, comprising 688 genes showing upregulation and 461 genes displaying downregulation (Figure 2B). Moreover, 46 DEGs overlapped between GSE72326 and GSE16134 (Figure 2C).

### 3.2 WGCNA analysis

**FIGURE 3:**
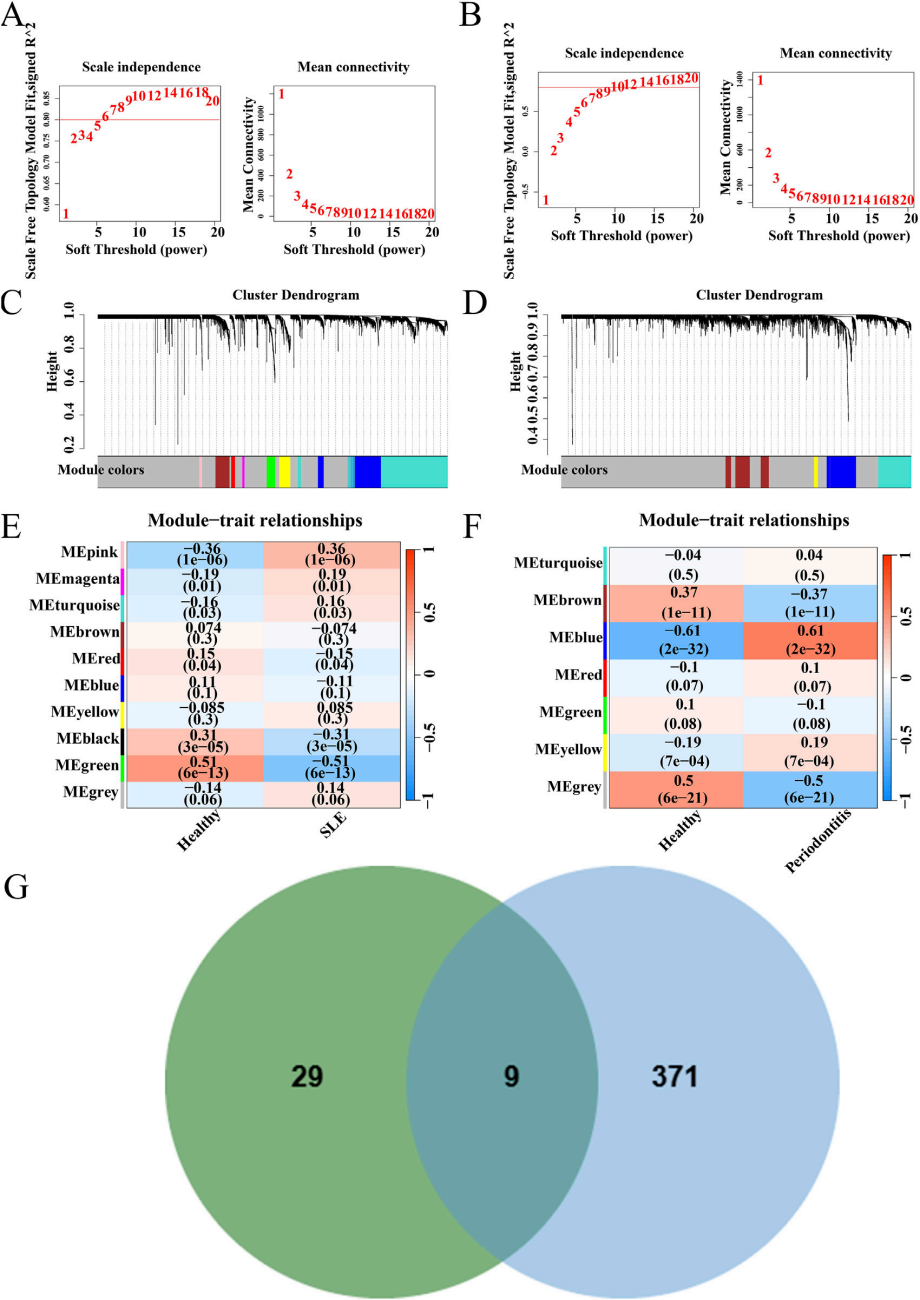
WGCNA of GSE72326 and GSE16134 datasets. **(A)** Network topology analysis results for dataset GSE72326. **(B)** Network topology analysis results for dataset GSE16134. **(C)** Gene modules and clustering results for dataset GSE72326. **(D)** Gene modules and clustering results for dataset GSE16134. **(E, F)** Heatmap depicting the correlation between module genes and diseases. **(G)** Genes overlapping in positively correlated modules.

WGCNA analysis was conducted on the datasets GSE72326 and GSE16134, respectively. Optimal soft-thresholding values of 14 and 18 were determined for GSE72326 (Figure 3A) and GSE16134 (Figure 3B), respectively. GSE72326 revealed 10 modules, whereas GSE16134 identified 7 modules (Figures 3C, D). Subsequently, each module was assessed for its association with the disease. In the GSE72326 dataset, the pink module exhibited the strongest association with SLE (r = 0.36, p = 1e-06), comprising 38 genes (Figure 3E). In the analysis of PD using the GSE16134 dataset, the blue module demonstrated the most significant positive association (r = 0.61, p = 2e-32), containing 380 genes (Figure 3F). Nine genes overlapped between the positively correlated modules of SLE and PD (Figure 3G). Additionally, these genes are closely associated with the pathogenesis of both diseases, prompting further in-depth research.

### 3.3 Functional analysis of shared genes

Nine genes overlapped between the modules of SLE and PD, with 46 genes shared among the DEGs. DEGs and module genes were combined as candidate genes for further analysis. We obtained a total of 50 candidate genes from DEGs and module genomes, which underwent further analysis.

**FIGURE 4:**
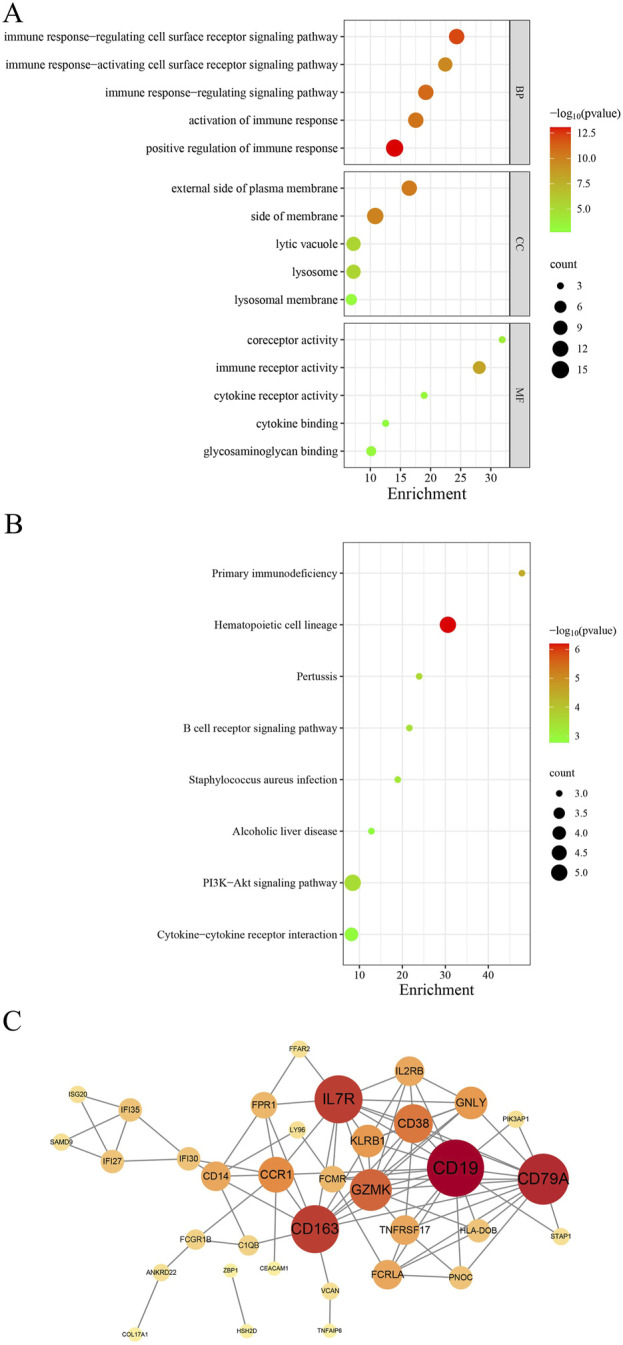
A comprehensive analysis of the shared genes was conducted. **(A)** GO enrichment analysis. **(B)** KEGG signaling pathway. **(C)** PPI network.

GO analysis demonstrated that BP were significantly associated with the upregulation of immune response, regulation of immune receptor signaling pathways, and immune signaling pathway regulation. MF were significantly associated with immune receptor activity, co-receptor activity, and cytokine receptor activity. CC was closely associated with the external side of the plasma membrane, membrane, lysosomal vacuole, and lysosome (Figure 4A).

The KEGG pathway analysis revealed that these shared genes were predominantly enriched in primary immunodeficiency, the PI3K-Akt signaling pathway, and the B cell receptor signaling pathway (Figure 4B).

We imported these 50 genes into the STRING database to elucidate their interactions. The analysis yielded 85 nodes and 114 edges, with a combined score exceeding 0.4 (Figure 4C). The top five ranked genes were B lymphocyte antigen CD19 (CD19), B lymphocyte antigen CD79A (CD79A), Cluster of Differentiation 163 (CD163), Interleukin 7 receptor (IL7R), and Granzyme K (GZMK).

### 3.4 Identification of potential shared hub genes through machine learning

**FIGURE 5:**
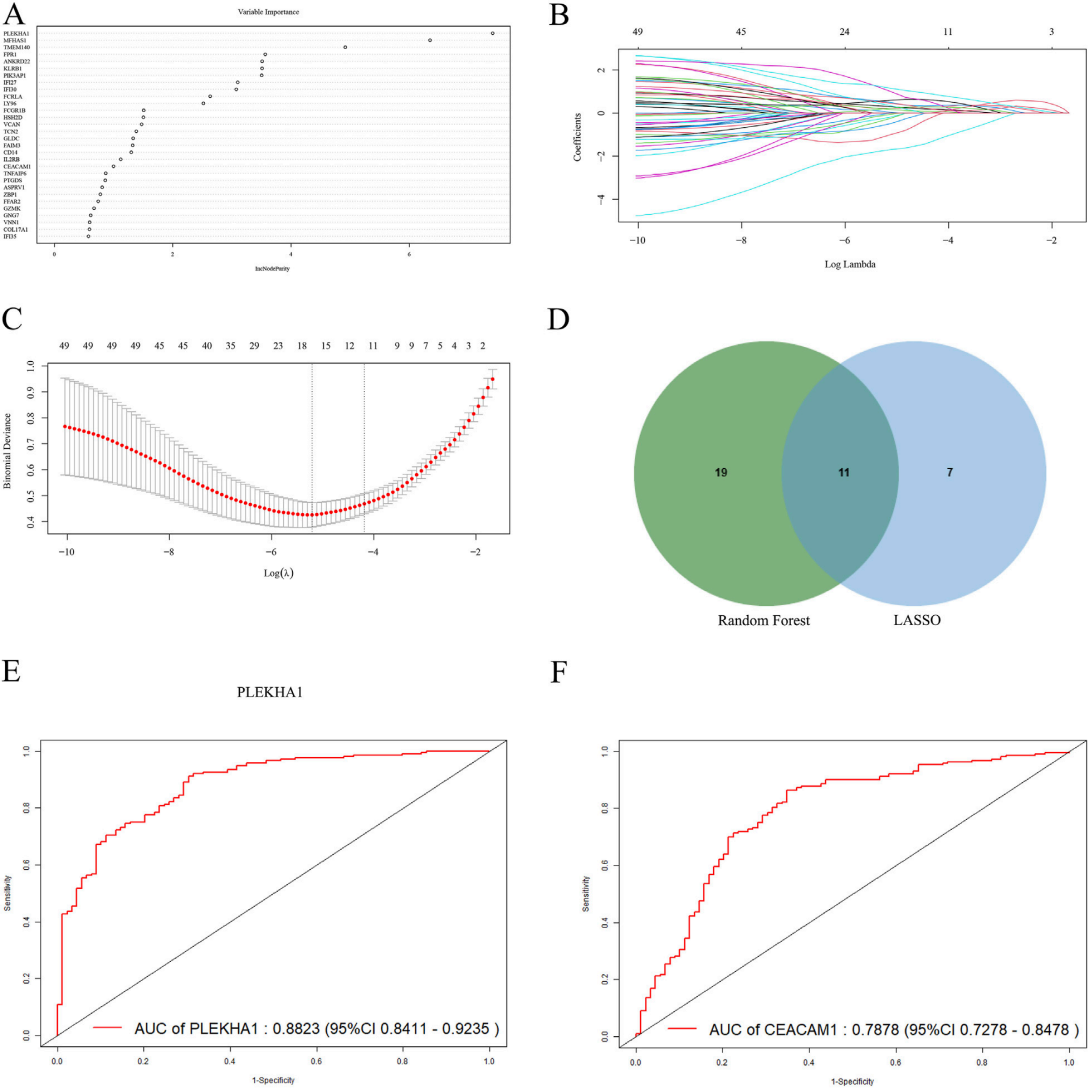
Screening and validating diagnostic biomarkers for SLE and PD. **(A)** The thirty most significant genes identified using the RF algorithm. **(B, C)** Cross-validation plot and coefficient contraction plot of LASSO regression analysis. **(D)** The intersecting genes selected by the RF and LASSO algorithms. **(E, F)** The ROC curves of PLEKHA1 and CEACAM1.

To further identify core genes with the highest diagnostic value, we conducted RF and LASSO regression analyses. From the 50 core genes, we selected the top 30 most significant genes for presentation (Figure 5A), with PLEKHA1, MFHAS1, and TMEM140 showing the highest MeanDecreaseGini values. Moreover, LASSO logistic regression identified 18 genes among the 50 core genes (Figures 5B, C). Upon intersecting the genes common to both the RF and LASSO analyses, we identified 11 hub genes shared between them (Figure 5D), which we deemed to hold significant diagnostic value. Subsequently, we used the new datasets GSE81622 and GSE10334 to evaluate the diagnostic performance of these hub genes using ROC curves (Table 1). We excluded three genes with AUC values below 0.6: FCGR1B (0.598), GZMK (0.5777), and IFI27 (0.4837). Among the remaining genes, PLEKHA1 (AUC = 0.882) and CEACAM1 (AUC = 0.788) had the highest AUC values, demonstrating a good ability to diagnose the risk of SLE with PD (Figures 5E, F).

**TABLE 1:** AUC of 11 hub genes.

Gene	AUC of ROC	95% CI
PLEKHA1	0.882	0.841–0.923
CEACAM1	0.788	0.728–0.848
TNFAIP6	0.731	0.668–0.794
TCN2	0.706	0.637–0.775
GLDC	0.658	0.577–0.739
GNG7	0.646	0.564–0.727
LY96	0.637	0.556–0.717
VCAN	0.603	0.523–0.683
FCGR1B	0.598	0.516–0.680
GZMK	0.578	0.492–0.663
IFI27	0.484	0.404–0.564

### 3.5 Immune infiltration analysis

**FIGURE 6:**
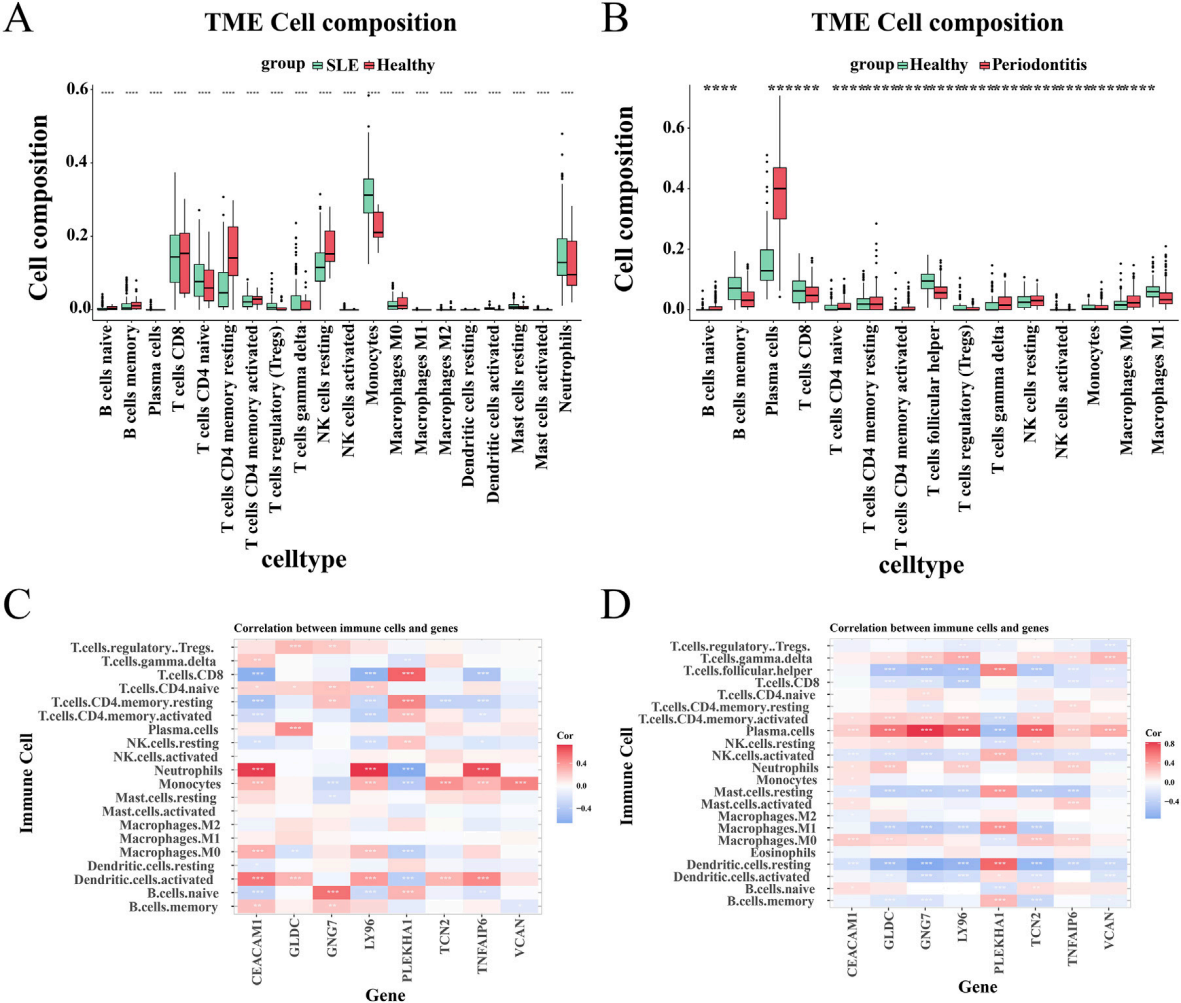
Analysis of immunoinfiltration. **(A, B)** The proportion of immune cells between patients with the disease and control groups. **(C, D)** The relationship between shared central genes and immune cells.

Next, we used the CIBERSORTx method to analyze immune cell infiltration in the SLE dataset GSE72326 and the PD dataset GSE16134, assessing differences between the disease and healthy groups. The violin plot showed a marked rise in monocytes and neutrophils among SLE patients relative to controls, while CD4 memory-activated T cells and resting NK cells significantly decreased (Figure 6A). PD patients exhibited significant increases in plasma cells, γδ T cells, M0 macrophages, and resting NK cells, while helper T follicular cells and CD8^+^ T cells markedly decreased (Figure 6B). In addition, we examined the relationship between eight shared core genes and immune cell composition. Heatmap results demonstrated positive correlations between neutrophils, monocytes, and activated dendritic cells with CEACAM1, LY96, and TNFAIP6 in SLE, while showing negative correlations with GNG7 and PLEKHA1 (Figure 6C). In patients with PD, plasma cells and γδ T cells were positively correlated with GLDC, GNG7, LY96, TCN2, TNFAIP6, and VCAN, and negatively correlated with PLEKHA1 (Figure 6D).

## 4 Discussion

SLE is a multifaceted autoimmune disorder, and its pathophysiological mechanisms remain incompletely understood (Hoi et al., 2024). PD is an infectious disease marked by an imbalance of microbial flora in periodontal tissues, resulting in chronic inflammation and gradual deterioration of periodontal supporting tissues. Recent studies indicate that individuals with SLE face an elevated risk of developing PD, with a risk approximately 1.76–1.78 times higher than the general population (Rutter-Locher et al., 2017; Bolstad et al., 2022). Furthermore, various biological hypotheses propose comparable pathophysiological mechanisms between these conditions, encompassing shared genetic, microbiological, immunological, and environmental risk factors (Sojod et al., 2021). Nevertheless, research investigating the genetic link between SLE and PD remains limited. Thus, the discovery of biomarkers and underlying molecular mechanisms pertaining to both SLE and PD via bioinformatics analysis holds significant clinical relevance.

This study utilized comprehensive bioinformatics analysis employing machine learning algorithms. Initially, we analyzed DEGs from the GSE72326 and GSE16134 datasets. Subsequently, DEGs were integrated with genes in the WGCNA module, leading to the identification of 50 candidate genes shared across the datasets. These 50 common candidate genes underwent enrichment analysis, revealing significant associations with the PI3K-Akt signaling pathway and B-cell receptor signaling pathway. Afterwards, we identified five core genes (CD19, CD79A, CD163, GZMK, IL7R) through PPI network analysis. To enhance the screening of diagnostic genes, machine learning algorithms were utilized to analyze overlapping genes. Ultimately, we identified 11 shared hub genes and validated them using ROC curves.

Our findings indicate a significant involvement of the PI3K-Akt signaling pathway in both the onset and progression of SLE and PD. It is crucial in regulating cell proliferation, differentiation, B cell and T cell receptor signaling, as well as macrophage polarization (Nestle et al., 2005; Dong et al., 2019; Linton et al., 2019; Chen et al., 2020). Similarly, the pathogenesis of SLE involves abnormal B cell activation, T cell dysregulation, and polarization of M1 macrophages (Rawlings et al., 2017; Ma et al., 2019; Tenbrock and Rauen, 2022). B-cell activating receptors such as BCR, CD40, and TLRs are all linked to the PI3K signaling pathway (Okkenhaug and Vanhaesebroeck, 2003; Donahue and Fruman, 2004; Hodson and Turner, 2009). In macrophages, the PI3K signaling pathway regulates the polarization state by modulating metabolic and inflammatory responses. Activation of this pathway suppresses M1-type macrophage polarization and enhances M2-type macrophage polarization (Long et al., 2021; Acosta-Martinez and Cabail, 2022). In clinical practice, focusing on the PI3K-Akt signaling pathway has also shown favorable therapeutic effects in patients with SLE (Shen et al., 2022). For instance, azithromycin can activate M2 macrophages via this pathway, thus reducing inflammation and improving the condition of SLE (Wang et al., 2018). The PI3K-Akt pathway plays a pivotal role in immune imbalance, inflammation regulation, and the maintenance of periodontal tissue homeostasis in PD. Studies have demonstrated that FBLN3 is upregulated in PD tissues, promoting M1 macrophage polarization via the EGFR/PI3K/AKT signaling pathway. This activation enhances the pro-inflammatory response and exacerbates periodontal tissue damage (Mu et al., 2024). Furthermore, PI3K-Akt modulates macrophage metabolism through HIF-1α-mediated glycolysis, sustaining M1 macrophage activation while suppressing M2 macrophage transition within the PD immune microenvironment (Zeng et al., 2024). Beyond immune cell polarization, PI3K-Akt plays a central role in the trained immunity of gingival fibroblasts (GFs). Porphyromonas gingivalis-derived lipopolysaccharide (LPS) has been shown to induce a trained immune response in GFs via PI3K/AKT pathway activation, leading to increased IL-6 and TNF-α secretion and sustaining inflammation through epigenetic modifications (Liu et al., 2024). Moreover, PI3K-Akt regulates osteoclast function, thereby influencing alveolar bone resorption and regeneration. Studies have identified the PI3K/AKT/GSK-3β signaling pathway as a key regulator of LPS-induced osteoclast activation and alveolar bone loss. Inhibiting this pathway has been found to reduce bone resorption, mitigate inflammation, and improve inflammatory bone loss (Lv et al., 2025). Additionally, PI3K-Akt may alleviate oxidative stress by enhancing cellular antioxidant capacity, thus contributing to the preservation of periodontal tissue homeostasis (Fu et al., 2023). In conclusion, the dysregulated activation of PI3K-Akt in PD not only influences immune cell polarization but also perpetuates inflammation and promotes alveolar bone loss by modulating trained immunity and bone metabolism. Given its critical role in both PD and immune dysregulation in SLE, PI3K-Akt represents a promising therapeutic target. Our findings further corroborate its pathological significance in PD and provide supporting evidence for its potential in targeted therapy.

In our research findings, hub genes are equally crucial for the clinical significance of both SLE and PD. We have identified eight key hub genes as diagnostic biomarkers (PLEKHA1, CEACAM1, TNFAIP6, TCN2, GLDC, GNG7, LY96, VCAN). Based on the current research status, we have focused on VCAN and LY96. While direct interactions between VCAN, LY96, and SLE remain undisclosed in current studies, their potential associations with SLE warrant attention. The VCAN gene encodes the Versican protein, a binding protein crucial for tissue morphogenesis and extracellular matrix formation, which is integral to tissue inflammation in response to infection and tissue damage (Wight et al., 2014). Versican exerts pro-inflammatory effects by modulating the adhesion of myeloid and lymphoid cells, particularly T lymphocytes and monocytes, which are regulated by multifunctional proteoglycans (Gill et al., 2010; Potter-Perigo et al., 2010; Evanko et al., 2012). Moreover, Versican interacts with inflammatory cells through two pathways: indirect binding to hyaluronic acid, and direct interaction with receptors like CD44, P-selectin glycoprotein ligand-1 (PSGL-1), and toll-like receptors (TLR), found on both immune and non-immune cell surfaces. These engagements activate signaling cascades that stimulate the synthesis and secretion of inflammatory cytokines, such as TNF-α, IL-6, and NF-κB (Wight et al., 2020). Notably, CD44 stands out as a significant therapeutic target in SLE, as it governs disease progression by modulating B cell activation and proliferation (Yi et al., 2023). Reduced expression of PSGL-1 in neutrophils of active SLE patients may lead to excessive release of Neutrophil Extracellular Traps (NETs). This excessive NETs release may exacerbate the disease by triggering an immune response through their DNA content, worsening inflammation and autoimmune reactions, thereby driving disease progression (Muñoz-Callejas et al., 2023). Additionally, the significance of VCAN in PD warrants attention. Expression levels of TLR4 are markedly elevated in PD patients, positively correlating with disease severity (Promsudthi et al., 2014). LY96, also known as myeloid differentiation factor-2 (MD-2), plays a crucial role in the innate immune response by serving as a ligand receptor tightly bound to (TLR4) (Wu et al., 2022b). TLR4 is involved in the pathogenesis of SLE by regulating the inflammatory response and promoting autoantibody production. Inhibiting TLR4 can alleviate inflammation and ameliorate kidney damage (Ma et al., 2018). Furthermore, TLR4 is implicated in immune response regulation during SLE pathogenesis through diverse pathways, encompassing immune cell activation, autophagy modulation, and interaction with type I IFN signaling pathway (Wu et al., 2022a). Thus, LY96 serves as the primary mediator in linking TLR4 with the association between SLE and PD. In summary, our findings suggest that VCAN and LY96 could represent novel therapeutic targets for both SLE and PD, thereby providing a robust groundwork for elucidating their underlying mechanisms in subsequent studies. Another important finding is that GNG7 demonstrates distinct correlations in SLE and PD, potentially influenced by variations in the immune microenvironment, signaling pathway regulation, and data sources. SLE is typically characterized by widespread immune dysregulation, whereas PD manifests as localized chronic inflammation. These contrasting immune landscapes may result in differences in the regulatory mechanisms of GNG7-mediated signaling pathways in the two diseases. Nevertheless, since CIBERSORTx immune infiltration analysis is applicable to various tissue environments, its findings remain biologically relevant to immune regulation, underscoring the need for further investigation into the underlying mechanisms. Future studies should employ single-cell transcriptomic analysis to elucidate the cell-type-specific roles of GNG7 and validate its regulatory mechanisms in immune cell function through *in vitro* experiments.

Additionally, alterations in immune cell populations appear to be pivotal in the interplay between SLE and PD. Our findings indicate that various immune cells are upregulated in SLE, including neutrophils and monocytes, while their expression is less pronounced in PD. Activated neutrophils are capable of generating a multitude of cytokines and chemokines, potentially leading to dysregulated functions of B and T cells, thereby exacerbating SLE pathogenesis (Smith and Kaplan, 2015). Aberrant monocyte activation in SLE stimulates dendritic cells and T/B cells through extensive interferon-alpha (IFN-α) and other inflammatory mediator secretion, culminating in autoantibody generation and tissue inflammation (Hirose et al., 2019). Conversely, In PD, substantial increases were observed in plasma cells, γδ T cells, M0 macrophages, and resting NK cells. The involvement of plasma cells is pivotal in the pathogenesis of PD, contributing to antibody production, immune response regulation, and inflammation-induced bone loss (Zouali, 2017). γδ T cells, abundant in epithelial tissues such as gingival tissues, may exert their functions in PD through diverse mechanisms, including immune surveillance, maintenance of immune homeostasis, and modulation of epithelial tissue repair processes (Figueredo et al., 2019). Notably, despite variations in the immune cell profiles upregulated in SLE and PD, there exists a close interconnection among these cells. Specifically, neutrophils support the survival and function of plasma cells by releasing B cell activating factor (BAFF) and directly interacting with them, thereby augmenting the immune response through enhanced plasma cell survival and antibody production (Zhao et al., 2019; Sabat et al., 2023). Additionally, γδ T cells modulate neutrophil activation and polarization by secreting IL-17, and encourage neutrophils to engage in suppressing T cell immune responses (Coffelt et al., 2015). In monocytes and γδ T cells, the v-γ9vδ2 + T cell subset within γδ T cells can activate monocytes by secreting inflammatory molecules like IFN-γ and TNF-α, inducing their adhesion and aggregation, and enhancing monocyte survival. Conversely, monocytes can induce the activation of Vγ9Vδ2+ T cells by accumulating and presenting phosphate antigens, thereby establishing a bidirectional regulatory interaction between these cell types (Chan et al., 2022). Monocytes critically contribute to the development and function of plasma cells by secreting factors vital for plasma cell survival and development, and by facilitating plasma cell proliferation through intercellular interactions (Mohr et al., 2009). The results of immune infiltration underscore the indispensable role of these immune cells in both SLE and PD.

## 5 Conclusion

In summary, our study clarifies the common molecular mechanisms underlying SLE and PD. We identified eight candidate genes (PLEKHA1, CEACAM1, TNFAIP6, TCN2, GLDC, GNG7, LY96, VCAN) as potential biomarkers for diagnosis. Analysis of immune infiltration demonstrated their intimate association with immune cells. Moreover, the PI3K-AKT signaling pathway likely plays a significant role in the interaction between SLE and PD. These discoveries establish a molecular basis for further investigating the relationship between SLE and PD.

## Data Availability

We confirm that the datasets can be found in the following online repositories. The names of the repositories and accession numbers are as follows: GSE72326: https://www.ncbi.nlm.nih.gov/geo/query/acc.cgi?acc=GSE72326, GSE16134: https://www.ncbi.nlm.nih.gov/geo/query/acc.cgi?acc=GSE16134, GSE81622: https://www.ncbi.nlm.nih.gov/geo/query/acc.cgi?acc=GSE81622, GSE10334: https://www.ncbi.nlm.nih.gov/geo/query/acc.cgi?acc=GSE10334.
